# Rethinking swimming performance tests for bottom-dwelling fish: the case of European glass eel (*Anguilla anguilla*)

**DOI:** 10.1038/s41598-020-72957-w

**Published:** 2020-10-02

**Authors:** P. Vezza, F. Libardoni, C. Manes, T. Tsuzaki, W. Bertoldi, P. S. Kemp

**Affiliations:** 1 International Centre for Ecohydraulics Research, Faculty of Engineering and Physical Sciences, University of Southampton, Southampton, S017 1BJ UK; 2 Department of Environment, Land and Infrastructure Engineering, Politecnicodi Torino, Torino, Italy; 3 Department of Civil, Environmental and Mechanical Engineering, University of Trento, Trento, Italy

**Keywords:** Freshwater ecology, Animal behaviour, Behavioural ecology

## Abstract

Systematic experiments on European eel (*Anguilla anguilla*) in their juvenile, early life stage (glass eel), were conducted to provide new insights on the fish swimming performance and propose a framework of analysis to design swimming-performance experiments for bottom-dwelling fish. In particular, we coupled experimental and computational fluid dynamics techniques to: (i) accommodate glass eel burst-and-coast swimming mode and estimate the active swimming time (t_ac_), not considering coast and drift periods, (ii) estimate near-bottom velocities (U_b_) experienced by the fish, rather than using bulk averages (U), (iii) investigate water temperature (T) influence on swimming ability, and (iv) identify a functional relation between U_b_, t_ac_ and T. Results showed that burst-and-coast swimming mode was increasingly adopted by glass eel, especially when U was higher than 0.3 ms^-1^. Using U rather than U_b_ led to an overestimation of the fish swimming performance from 18 to 32%, on average. Under the range of temperatures analyzed (from 8 to 18 °C), t_ac_ was strongly influenced and positively related to T. As a final result, we propose a general formula to link near-bottom velocity, water temperature and active swimming time which can be useful in ecological engineering applications and reads as ${\rm{U}}_{\rm{b}}=0.174\cdot \left({{\rm{t}}_{\rm{ac}}}^{-0.36}\cdot {\rm{T}}^{0.77}\right)$.

## Introduction

Fish capacity for swimming has profound ecological importance in determining survival. Swimming performance is a crucial factor that influences predator–prey interactions, reproduction, migration, habitat shifts and dispersal^1^. Fish employ different swimming modes ranging from nomadic cruising over long distances to rapid short bursts. In particular, Beamish^2^ identified three categories of fish swimming modes: sustained, prolonged and burst. Sustained swimming can be maintained for long periods (> 240 min, or > 200 min in Brett^3^) and is fueled aerobically. Prolonged swimming is also fueled aerobically but is of shorter duration than sustained (20 s—200 min) and results in fatigue. Burst swimming enable fish to quickly reach their highest speed through anaerobic respiration, but can be maintained only for short periods (< 20 s)^1^. Sustained and prolonged swimming modes are used by highly migratory species or those that must swim to maintain position in the water column. Burst swimming is used by fish to flee from predators or, during migration, to pass obstacles, such as man-made river-infrastructure, which cause abrupt increases in flow velocity. Within this context, man-made river-infrastructure, such as dams, weirs and culverts, may block or delay fish migration and contribute to population decline^4–7^. Therefore, to design and evaluate fish passes that enable fish to overcome such barriers, a reliable and ecologically-relevant measure of fish swimming performance is required^1,8–10^.

The swimming performance of several fish species has been quantified and compared using so-called swimming-curves (also called fatigue-curves) relating the time a fish can swim continuously against a stream and the velocity of the stream itself. Historically, these curves have been obtained from experiments conducted using either constant^2^ or incremental^1^ velocities while the fish is constrained in a swim chamber (i.e. a pressurized flow without free-surface), and forced to swim against the moving water. Under these conditions, fish tend to hold position against the current, until they reach exhaustion, namely the condition corresponding to fish being impinged against a downstream screen and unable to escape. The duration the fish can swim before exhaustion is recorded and related to the velocity of the flow. The constant (or fixed) velocity method requires the velocity to be kept constant throughout the experiment whereas the incremental method involves progressive increases in flow velocity after arbitrarily-chosen time spans. The constant velocity method is in general more time-consuming than the incremental velocity approach, but considered to be more straightforward and informative^10^, being less biased by differences in the chosen testing protocols. Indeed, fish swimming tests, carried out with incrementally-increased flow velocity, usually differ from one another in defining both incremental velocity steps (e.g., from 0.5 to 1 fish body length/s) and the prescribed duration for each increment (from 10 to 60 min^1^).

In general, the flow velocity experienced by fish during swimming tests is commonly assumed to be, implicitly or explicitly, identical to the average cross-sectional velocity. This average velocity value is estimated by dividing flow discharge by the channel cross-sectional area^1,11–13^, or by measuring velocities in one cross-section or at one single point in the channel^14,15^. Rarely^10,16,17^, the heterogeneity of the flow field is taken into consideration and measured. In channel flows and swim chambers, though, the velocity field is not homogeneous; a boundary layer always develops near the walls and the thickness of it varies according to the wall roughness, the Reynolds number and the streamwise distance over which the boundary layer develops (e.g., just after a flow straightener or a mesh screen). Several fish-endurance tests^14–16^ report that, very often, fish tend to swim close to the channel walls and corners for a large amount of time as they attempt to utilize lower velocity-regions and save energy^17,18^. Therefore, using an average velocity, and not taking into account variations in the flow field caused by solid walls, may lead to an overestimation of the velocity experienced by fish and, hence, the fish swimming performance.

Fluctuations in swimming velocity, and the relative power expenditure, are important additional components of swimming performance^16^. Depending on flow conditions, many species employ different swimming strategies, e.g., steady or continuous swimming at moderate speeds, sprint or burst-and-coast at high speeds^7,19,20^. Burst-and-coast swimming consists of alternating phases of active swimming and gliding and is used by fish to reduce power expenditure when swimming at high constant velocities^14,19,21^. These fluctuations in swimming velocity can be influenced by the experimental setup, with the inhibition of intermittent locomotion when small swim chambers relative to fish size are used, resulting in earlier fatigue of fish and conservative estimates of swimming performance^14^. Longer open channel flumes may be used to provide more realistic measures of swimming capability of unconstrained fish that are able to exhibit performance enhancing behaviors^19,22^. However, variability in fish swimming speed has received little attention when assessing swimming performance and has been poorly taken into consideration when quantifying the swimming-time during experiments^23,24^.

Water temperature strongly influences the physiology and swimming performance of fish^25^. A widely-accepted assumption is that performance traits, such as oxygen consumption, metabolic rate or locomotion, peak at an optimum temperature, and cease at some critical minimum and maximum threshold (fish thermal limits^26,27^). To a lesser extent, beside fish physiology, water temperature also influences water viscosity and hence the viscous drag-forces experienced by fish while swimming, which, in turn, influences the energetic costs for locomotion and, ultimately, swimming performance. This is particularly relevant for small fish or fish at larval life-stages^28^, for which viscous drag dominates over pressure drag. These temperature-effects on fish swimming performance have been mentioned as important, but, they are rarely taken into consideration (see e.g.^28–30^).

In this study, we investigate the swimming performance of European eel (*Anguilla anguilla*) in its juvenile, early life stage (glass eel). The European eel is a catadromous, bottom-dwelling fish that spawns in the Sargasso Sea, and as larvae (leptocephali) spend between one to three years drifting with currents across the Atlantic Ocean before metamorphosing into the transparent “glass eel” stage on reaching the European coast. On entering estuaries they continue to metamorphose into pigmented elvers and embark on an upstream migration until they reach a place of residence and become yellow eel. The European eel is a critically endangered species; stocks have declined by 90–99% since the 1980s^31^, and one of the key reasons for this is the fragmentation of rivers caused by the installation of man-made structures in many water-courses^4^. To design effective solutions that allow glass eel to by-pass such structures, it is crucial to have reliable and quantitative information on their swimming performance in moving water^32^.

Previous studies of the swimming performance of juvenile European eel^12,23,33–36^ provide inconsistent results^15,35^. Clough and Turnpenny^33^ proposed a burst velocity value equal to 0.41 ms^-1^, that is lower than the value proposed by McCleave^23^, which is 0.54 ms^-1^, or the value of 0.80 ms^-1^ provided by Tsukamoto et al.^12^. Solomon and Beach^35^ argued that these inconsistencies may be explained by juvenile eel body-size and water temperature effects. Nonetheless, as for other fish species, fish swimming curves of glass eel have been derived using average cross-sectional velocities in the flume and total-time to fatigue, without taking into account their swimming position during these tests and burst-and-coast swimming behavior. These can all be considered as significant shortcomings because it has been observed that juvenile eel tend to swim in the near-bed flow regions^6,23,32^, and may display alternating phases of active swimming and gliding^23^.

To quantify the swimming performance of glass eel, our study reports swimming curves that were derived by processing data obtained from flume experiments and Computational Fluid Dynamics (CFD) simulations. Results are presented to identify to what extent swimming curves depend on: (i) active swimming time (i.e. the total time fish actually swam, excluding coast and drift periods) versus total-swimming time to fatigue; (ii) flow velocity experienced by the fish in the near-wall versus average cross-sectional velocities; and (iii) water temperature during experiments. The results are then discussed and elaborated to identify and explain (iv) the observed scaling-relation that link flow velocity, time to fatigue and water temperature for glass eel.

All these findings provide a rigorous framework of analysis that can be employed as a benchmark to design new experiments for bottom-dwelling fish and a general formula to be employed in ecological engineering applications, such as the evaluation of the migration potential of glass eel in rivers^32^ and the development of Agent Based Models to predict glass eel dispersal in river networks.

## Results

**Figure 1:**
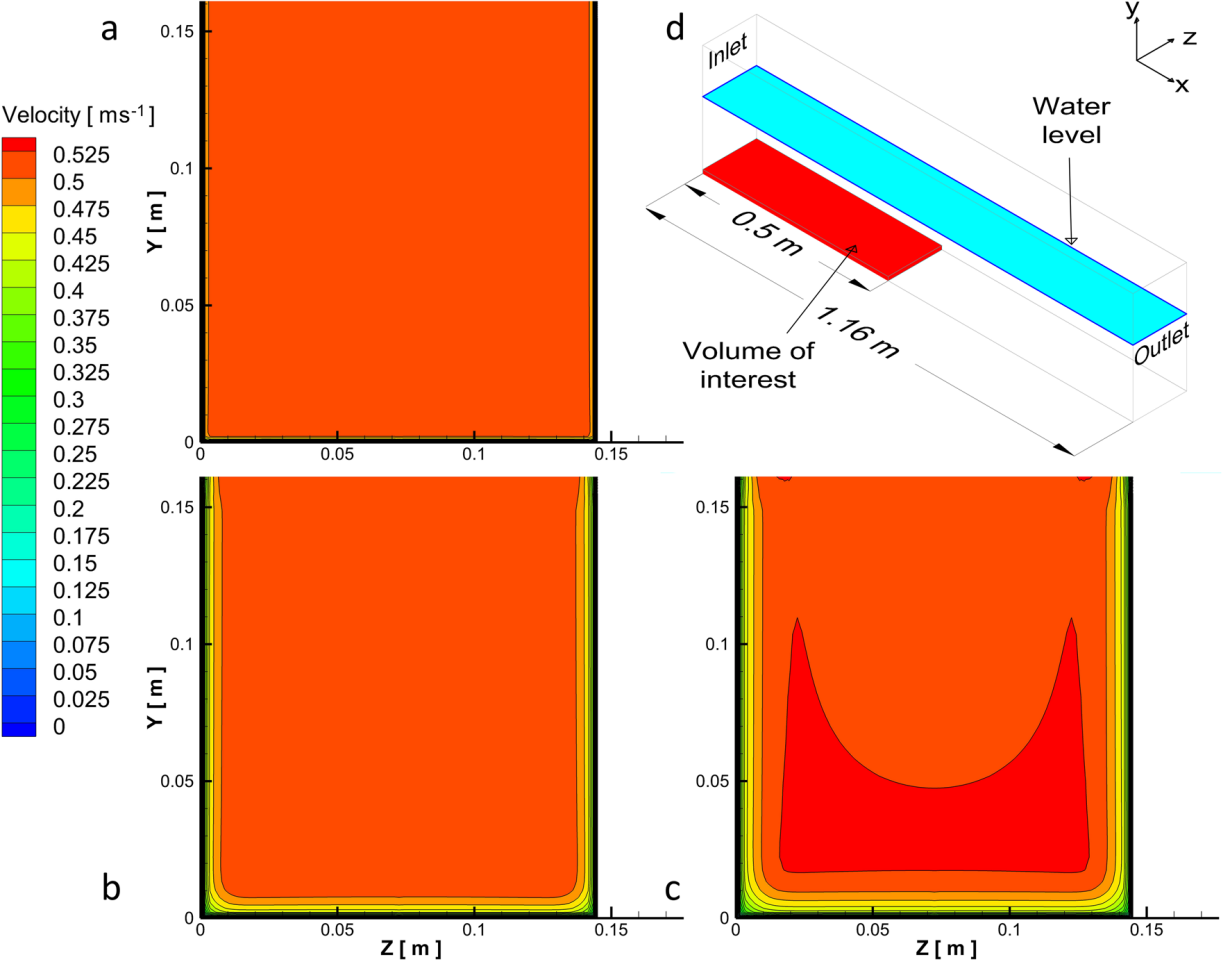
Velocity magnitude using the CFD k-ε model for U = 0.5 ms^−1^ condition at a distance of **(a)** 0.04 m, **(b)** 0.29 m, and **(c)** 0.44 m from the upstream screen. Volume of interest **(d)**, highlighted in red, used to estimate swimming velocities; the selected rectangular cuboid has a length 0.5 m, height 0.003 m and equivalent width as the channel.

From the CFD model, the flow fields were, on average, fairly homogeneous over the vast majority of the channels’ cross-section, with exception of the near-walls flow region which was subjected to the presence of a boundary layer, whose thickness increased with increasing downstream distance from the screen (Fig. 1). For the vast majority of the time, eel swam: (i) near the channel bed (on average over 91% of the active swimming time, SD =  ± 8%), (ii) within the first 50 cm of the upstream section of the channel (on average over 93% of the active swimming time, SD =  ± 6%), and (iii) across the channel bed from one channel side-wall to the other. Glass eel were thus observed to swim primarily in a specific “control volume” (highlighted in red in Fig. 1d), defined as 50 cm long, 3 mm high (equivalent to the average body thickness of the glass eel used in the experiments) and over the whole width of the channel. The large majority (95%) of the tested eel swam for less than 30 min (upper time-threshold set for each trial). Considering this upper time-threshold, values of 30 min were observed only in a few treatments, when water temperature was equal to 15 °C and 18 °C, thus not influencing the median value of swimming times used in the analysis.

### Active swimming time

**Figure 2:**
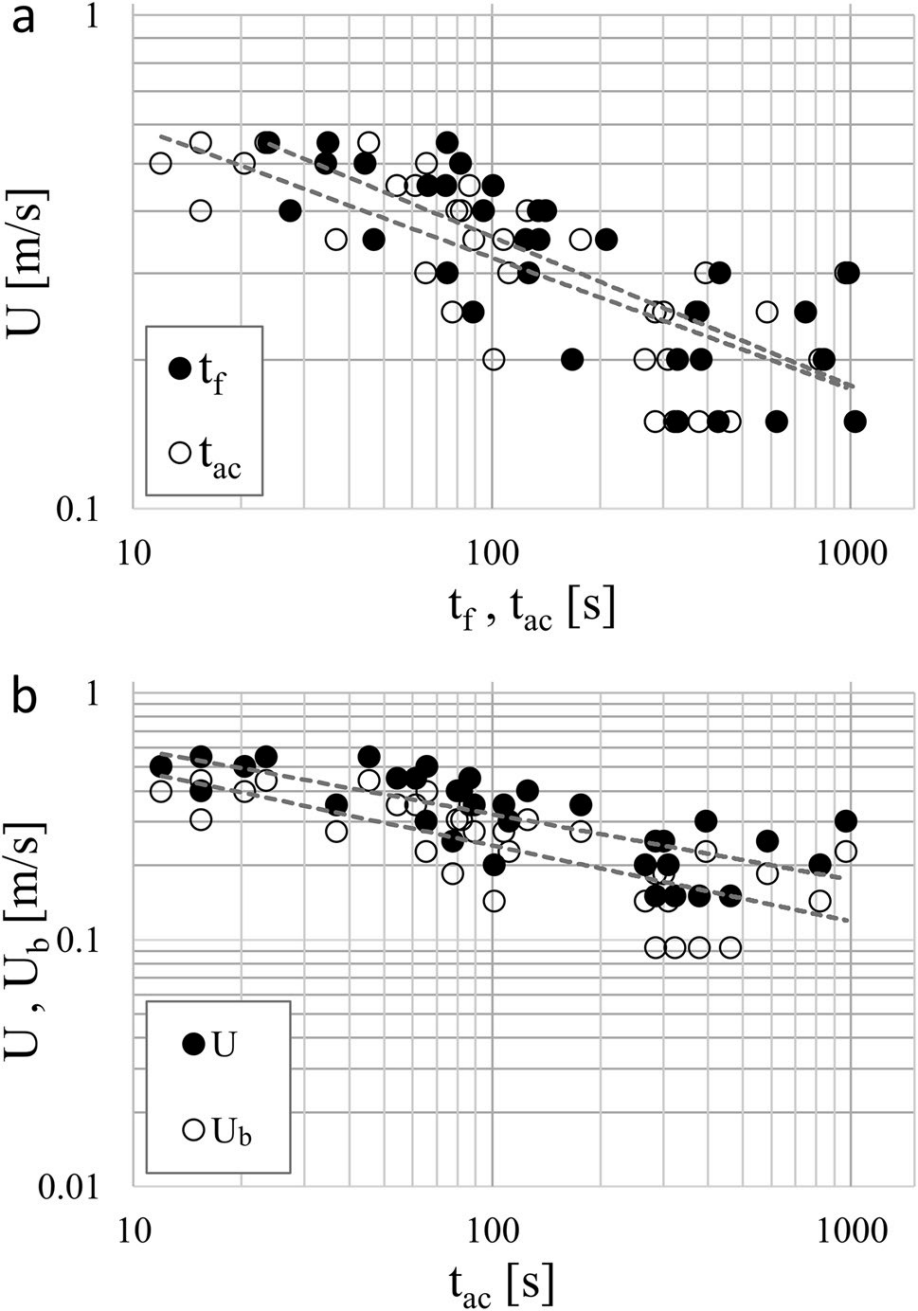
Swimming performance data (symbols) and curves (dashed-grey lines) expressed in log–log scale; t_f_, and t_ac_ are the total and active swimming time; U and U_b_ are the average cross-sectional velocity and the near-bottom velocity, respectively. Note that in both panels **(a)** and **(b)**, the influence of temperature on swimming performance is not considered, with data aggregated for all temperature treatments. Swimming curves reported in panel **(b)** are expressed as $\rm{U}=1.099\cdot {{\rm{t}}_{\rm{ac}}}^{-0.266}$ and ${\rm{U}}_{\rm{b}}=0.990\cdot {{\rm{t}}_{\rm{ac}}}^{-0.307}$.

Burst-and-coast swimming strategy was increasingly observed when average cross-sectional velocity increased (Fig. 2a). Slight significant difference (t-test, p = 0.09) between total (t_f_) and active (t_ac_) swimming times was observed for U > 0.3 ms^-1^. This implies that the swimming curves pertaining to t_f_ and t_ac_ (dashed-grey lines) can be considered different in terms of both intercept and slope. Since t_ac_ is a more rigorous estimate of the actual swimming time and the total power used by eel when holding their position against a current, data pertaining to t_f_ were disregarded for further analysis.

### Velocity experienced by the fish in the near-wall region

Near-bottom velocity (U_b_) experienced by fish was calculated by averaging computed velocities from the CFD model. In the water volume of interest, U_b_ was lower than its bulk counterpart (U) (Fig. 2b). A significant difference in intercept (t-test, p < 0.05) between the swimming curves pertaining to the two different velocities was evident and quantified to be in the range 18–32%. The slope of the two swimming curves pertaining to t_f_ and t_ac_ were similar (t-test, p >> 0.10) and equal to -0.3 and -0.27, respectively. Since glass eel swam near the channel bed, and the difference between U_b_ and U was significant, swimming-curves were calculated using U_b_ and not the average cross-sectional velocity U.

### Scaling-relation among flow velocity, time to fatigue and water temperature

**Figure 3:**
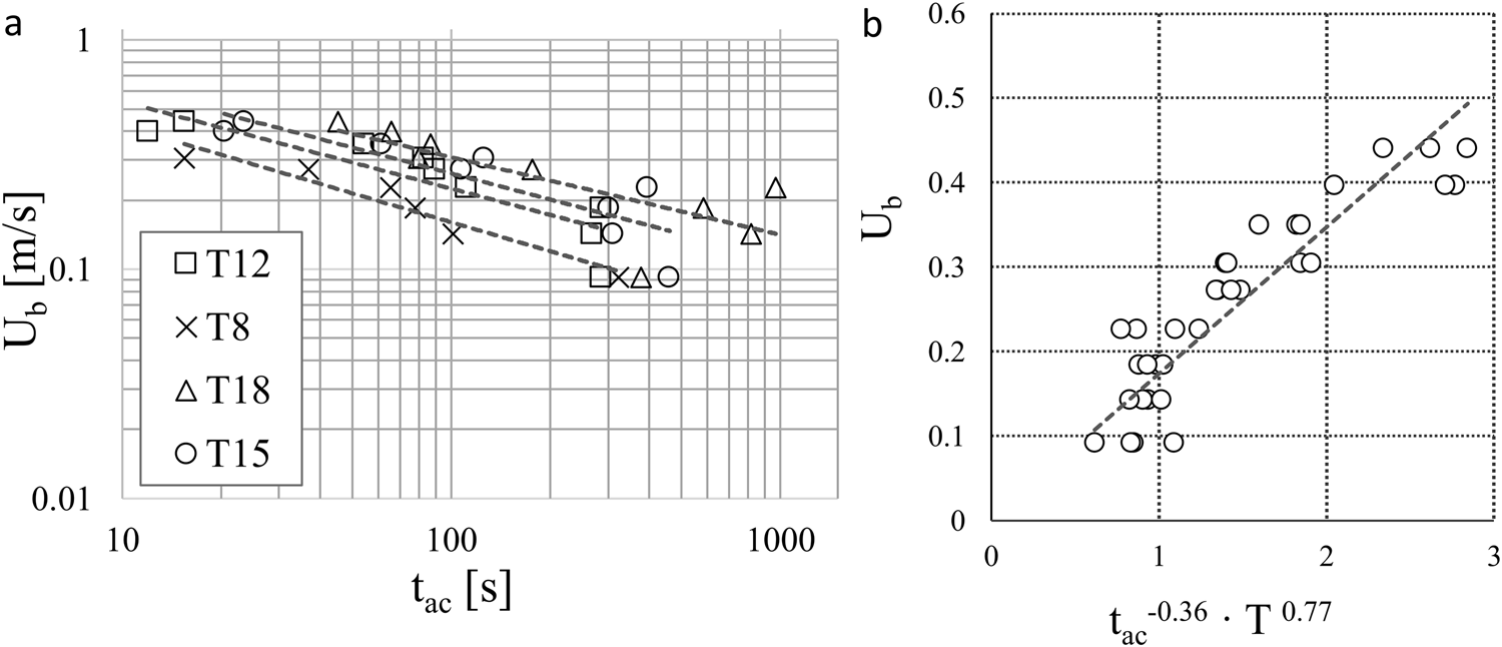
Swimming curves for different water temperatures. Regression lines represent **(a)** swimming curves obtained at different temperatures T = 8, 12, 15, 18 °C, and **(b)** the functional relationship among flow velocity at the channel bed (U_b_), the active fish swimming time (t_ac_) and water temperature (T) as outlined by Eq. (1). Swimming curves reported in panel **(a)** are expressed as ${\rm{U}}_{\rm{b}}=1.113\cdot {{\rm{t}}_{\rm{ac}}}^{-0.421}$, for T = 8 °C; ${\rm{U}}_{\rm{b}}=1.295\cdot {{\rm{t}}_{\rm{ac}}}^{-0.380}$, for T = 12 °C; ${\rm{U}}_{\rm{b}}=1.486\cdot {{\rm{t}}_{\rm{ac}}}^{-0.377}$, for T = 15 °C; ${\rm{U}}_{\rm{b}}=1.464\cdot {{\rm{t}}_{\rm{ac}}}^{-0.338}$, for T = 18 °C.

Swimming curves as relations between U_b_ and t_ac_ were computed for each temperature (Fig. 3a), showing a strong influence by and a positive relation to water temperature. Swimming curves showed high coefficient of determination (R^2^) equal to 0.92, 0.78, 0.76 and 0.60 for treatments at temperatures T = 8, 12, 15, 18 °C, respectively. Although there was no difference (t-test, p >> 0.10) between the estimated power-law slopes, (equal to 0.421, 0.380, 0.377, 0.338 for T = 8, 12, 15, 18 °C, respectively) the intercepts significantly differed between trials conducted at 8 °C and 12 °C, 8 °C and 15 °C, 8 °C and 18 °C and between 12 °C and 18 °C (t-test, p < 0.05). Furthermore, no difference in terms of intercepts was observed between trials conducted at 12 °C and 15 °C, or between those at 15 °C and 18 °C (t-test, p >> 0.10).

Fitting a multiple regression line, using the near-bottom velocity (U_b_) experienced by glass eel as dependent variable (y-var), and the active fish swimming time (t_ac_) and water temperature (T) as independent variables (x-var), led to the identification of a mathematical relation among the three physical quantities. The multiple regression exponents were computed as -0.36 and 0.77 for t_ac_ and T, respectively, with a coefficient of determination R^2^ = 0.81. The obtained function $f({{\rm{t}}_{\rm{ac}}}^{-0.36}\cdot {\rm{T}}^{0.77})$ was linearly related to U_b_ as follows:

1 $${\rm{U}}_{\rm{b}}=0.174\cdot ({{\rm{t}}_{\rm{ac}}}^{-0.36}\cdot {\rm{T}}^{0.77}).$$

## Discussion

The ability to accurately estimate swimming performance is crucial to predict whether river infrastructure is likely to negatively impact fish movement (see e.g.^25^). This is particularly relevant for the conservation of endangered species, including the European eel that strongly depends on longitudinal movements between the ocean and rearing habitat within rivers and streams. As eel are catadromous, it is the juvenile life-stage that embarks on the upstream migration, and, due to their small size, the rate and extent of their movement is restricted by their swimming performance and ability to negotiate in-stream barriers such as weirs and barrages^15^.

Current approaches used to estimate the swimming performance of glass eel suffer from several major limitations, including the selection of representative flow velocities and accommodation of temperature effects on fish swimming time-to-fatigue. Specifically, the commonly-employed average cross-sectional velocity is not representative of the hydrodynamics experienced by glass eel, since this fish swims close to the channel bed, where the flow velocity is lower. Moreover, water temperature may have an important influence on swimming performance, which generally peaks at an optimum temperature, and ceases at some critical minimum and maximum threshold. To address these shortcomings, the present study integrated constant velocity swimming-performance tests with Computational Fluid Dynamics (CFD) to facilitate the reliable estimation of the velocities experienced by eel in the near-wall region. The developed CFD model was relatively simple to calibrate and allowed for the construction of the reported swimming curves. The k-ε closure method permitted a time efficient modelling process to be adopted that enabled systematic exploration of the velocity magnitude in the spatial domain with excellent resolution. To our knowledge, this is the first time CFD tools are used to provide velocity data that are integrated with the results of experiments on swimming performance of fish.

The dimensions of the channel used to test fish swimming performance can have an important influence on swimming performance curves. McCleave^23^ studied the swimming activity of juvenile European eel (mean fish length, 7.2 cm, ranging from 6.9 to 7.5 cm) in a darkened, rectangular swimming chamber. The average cross-sectional velocity U ranged from 0.25 to 0.5 ms^-1^, whereby the total swimming time-to-fatigue decreased from 146 to 16 s, respectively. The water temperatures in that study (ranged from 11.1–13.3 °C) were quite similar to our 12 °C treatment, but the overall performance was greater in our study, with total swimming time (t_f_) at U = 0.25 and 0.5 ms^-1^ being approximately 220 and 40 s, respectively (based on regression analysis), despite the almost-identical mean fish length. The key difference between the two studies was the length of swimming area, which was 1.16 m in our study (> 15 times the mean length of the fish), and 0.5 m in McCleave^23^. Likewise, cross-sectional area also appears to be influential. Based on tests using a swimming length of 1.8 m, Clough and Turnpenny^33^ measured the swimming performance of juvenile eel through a narrow, circular Perspex pipe (0.04 m diameter). Using the developed swimming curve in Clough and Turnpenny^33^, the burst swimming velocity (total swimming time equal to 20 s) for a 7.0 cm long juvenile eel at a water temperature of 11.1 °C was 0.41 ms^-1^. Based on the results of our study conducted at 12 °C, we predict glass eel to be able to swim 70 s at an average flow velocity U = 0.41 ms^-1^. These comparisons may indicate the importance of both the length and the total volume of the working section when testing swimming performance in hydraulic facilities.

In our study, burst-and-coast swimming was increasingly observed for near-bottom velocities (U_b_) exceeding 0.2 ms^-1^(i.e., average cross-sectional velocity > 0.3 ms^-1^). This suggests that the burst-and-coast swimming mode is beneficial under higher velocities because intermittent swimming bestows energetic benefits. Indeed, it is well known that gait transitions, including burst-and-coast swimming, enables recovery and thus enhanced swimming performance^19,37^. Failure to provide sufficient test space can prevent the subject fish from displaying behaviors that can enhance performance, resulting in conservative estimates of swimming capability^19^.

The average cross-sectional velocity is clearly not representative of swimming conditions of bottom-dwelling fish, like Anguillidae, that gain energetic advantages by exploiting the low velocities characterizing near-wall flow regions. The present study demonstrates that, contextually to the flow conditions explored herein, differences in swimming-curve intercepts ranged between 18 and 32%. These differences are essentially equivalent to the average observed differences between U and U_b_, which can be explained with the following scaling arguments. In open channel flows, near-bottom velocities U_b_ scale with the friction velocity, ${\rm{u}}_{*}$(a fundamental scaling velocity equal to the square root of the shear stress, τ_0_, divided by the water density, i.e. ${\rm{u}}_{*}=\sqrt{{\uptau }_{0}/\uprho }$), which is related to the average velocity U via the Darcy-Weisbach friction factor $f$ as ${\rm{u}}_{*}=\rm{U}\sqrt{\frac{\rm{f}}{8}}$. The near bed velocities ${\rm{U}}_{\rm{b}}$ were averaged over a 3 mm thick volume of water, which embraces the so-called viscous sub-layer, the buffer sub-layer and, for some experimental conditions it may capture the logarithmic layer^38^. This is easy to demonstrate by scaling the height of the averaging volume (i.e. 3 mm) by means of the viscous length scale $\upupsilon/ {\rm{u}}_{*}$. This non-dimensional height reaches, at most, the value of 73 which is indicative of a flow region within the logarithmic layer. Therefore, within the averaging volume it is fair to state that ${\rm{U}}_{\rm{b}}\approx O\left(10{\rm{u}}_{*}\right)$ and hence^37^,

2 $${\rm{U}}_{\rm{b}}\propto {f}^{1/2}\rm{U}.$$

Note that the friction factor $f$ may depend on: (i) the Reynolds number (i.e., $\rm{Re}=\rm{U}4\rm{R}/\upnu$, where U is the average cross-sectional velocity, R is the hydraulic radius defined as the ratio between the wet area and the wet perimeter of the channel cross-section and ν is the water kinematic viscosity); (ii) the relative roughness of the flow; or (iii) both, if the flow is in the hydraulically-smooth, hydraulically-rough and -transition regime, respectively. In the present paper, experiments were carried out in the hydraulically-smooth regime, therefore we can assume $f=0.316{\rm{Re}}^{-1/4}$^39^ and hence, from Eq. (2) we obtain

3 $${\rm{U}}_{\rm{b}}\propto {(\rm{Re}}^{-1/8})\rm{U},$$

where the proportionality coefficient is of order 1. From Fig. 2b, it is possible to infer that the average velocity U can be expressed as

4 $$\rm{U}={\rm{\alpha }}_{1}{\rm{t}}_{\rm{ac}}^{{\beta }_{1}},$$

where ${\rm{\alpha }}_{1}=1.099$ and ${\beta }_{1}= -0.266$. Coupling Eqs. (3) and (4) leads to

5 $${\rm{U}}_{\rm{b}} \propto {\left(\frac{\rm{R U}}{\upnu }\right)}^{- \frac{1}{8}}{{\alpha }}_{1}{\rm{t}}_{\rm{ac}}^{{\upbeta }_{1}}= {\left(\frac{\rm{R}}{\upnu }\right)}^{- \frac{1}{8}}{\rm{U}}^{-\frac{1}{8}}{{\alpha }}_{1 }{\rm{t}}_{\rm{ac}}^{{\upbeta }_{1}}={\left(\frac{\rm{R}}{\upnu }\right)}^{- \frac{1}{8}}{{{\alpha }}_{1 }}^{\left( 1- \frac{1}{8}\right)}{\rm{ t}}_{\rm{ac}}^{\left(1 - \frac{1}{8}\right){\upbeta }_{1}}.$$

As observed in Fig. 2b, Eq. (5) demonstrates that referring to the near-bottom velocity U_b_ rather than the average cross-sectional velocity U, leads to a reduction of the intercept coefficient of a factor scaling as ${\left(\frac{\rm{R}}{\upnu }\right)}^{- \frac{1}{8}}{{\rm{\alpha }}_{1 }}^{\left(- \frac{1}{8}\right)}$, corresponding to a 24–26% reduction, which is very similar to that observed experimentally (i.e., 18–32%, see “Results” section). In addition, the exponent ${\upbeta }_{1}$ undergoes a reduction of about 1/8, i.e. 12.5%, which compares very well with the 14% variation of the power-law exponents associated with the swimming curves plotted in Fig. 2b and reported in the results section.

The active swimming time of glass eel (t_ac_) decreases with increasing near-bottom velocity and, for a given bottom velocity, t_ac_ increases with increasing water temperature (in the range 8–18 °C). This implies that, at higher temperatures, eel can sustain prescribed flow-velocities for longer times. Interestingly, for any temperature, U_b_ scales with t_ac_ as ${\rm{U}}_{\rm{b}}\sim {\rm{t}}_{\rm{ac}}^{\upbeta }$ with $\upbeta \cong -$ 1/3, on average. This may have some interesting implications which are now discussed.

It can be speculated that the flow resistance experienced by glass eel can be quantified as a drag force ${\rm{F}}_{\rm{D}}$ that scales as $\rho {\rm{C}}_{\rm{D}}a{\rm{U}}_{\rm{b}}^{2}$, where $\rho$ is the water density, ${\rm{C}}_{\rm{D}}$ is the fish drag coefficient and $a$ is the fish frontal area. The power used by glass eel (i.e. the energy spent per unit time) to hold their position against a current, can be expressed as $\rm{P}= \rho {\rm{C}}_{\rm{D}}\it{a}{\rm{U}}_{\rm{b}}^{3}$ and the total energy spent by the fish is therefore $\rm{E}={\rm{Pt}}_{\rm{ac}}=\rho {\rm{C}}_{\rm{D}}\it{a}{\rm{U}}_{\rm{b}}^{3}{\rm{t}}_{\rm{ac}}$. In the experiments reported herein, eel had an almost uniform body-size (i.e. a similar frontal area *a*) and, therefore, for a specific water temperature, $\rho$ and $a$ can be considered approximately as constant. For a prescribed water-temperature, the drag coefficient ${\rm{C}}_{\rm{D}}$ of the eel might retain some Reynolds number dependence (in general, the ${\rm{C}}_{\rm{D}}$ of a slender body immersed in a moving fluid reduces with increasing Re due to the weakening of viscous forces with respect to pressure forces in the total drag force experienced by the body), which translates, essentially, into a dependence on ${\rm{U}}_{\rm{b}}$. However, the range of Reynolds numbers experienced by glass eel in the experiments presented herein is too small to induce significant variations in ${\rm{C}}_{\rm{D}}$, which can therefore be considered, in good approximation, as constant. Therefore, it can be also reasonably assumed that the energy spent by the eel during a fatigue experiment, scales as $\rm{E}\sim {\rm{U}}_{\rm{b}}^{3}{\rm{t}}_{\rm{ac}}$. However, since the near-bottom velocity scales approximately as ${\rm{U}}_{\rm{b}}\sim {\rm{t}}_{\rm{ac}}^{-1/3}$ (Fig. 3a), it follows that the energy spent by the eel is equal to a constant which is a function of temperature only. This suggests that, for a specific water temperature, the energy spent by a fish in a fatigue test is constant and independent on flow intensity levels (i.e. ${\rm{U}}_{\rm{b}}$) or, in other words, this means that the swimming performance of glass eel might be energy-limited. Clearly, this hypothesis needs to be further substantiated by more experimental work allowing for direct measurement of oxygen (and hence energy) consumption during fatigue tests, possibly carried out using the framework of analysis presented herein. It could be also important to find an experimental, non-invasive technique able to track transparent glass eel while moving in flumes or swimming chambers (e.g.^40,41^). This will allow a better understanding of the link between swimming speed variability at constant flow and energy consumption^16^.

In the analyzed range of water temperatures (which represents common temperatures experienced by glass eel during upstream migration^32^) and flow velocities (Fig. 3b), Eq. (1) can be used to design a fish-pass or to evaluate its effectiveness for glass eel migration in a prescribed river reach. For instance, Vowles et al.^42^ proposed eel tiles as a cost-effective solution for mitigating the impacts of anthropogenic barriers to juvenile eel migration. Equation (1) can be used to verify whether velocities in the fish-pass are in an acceptable range, depending on the flow stage and the length of the eel tiles. Equation (1) provides also an estimate of the time needed by glass eel to circumvent dams and weirs or possible delays during migration. However, care would be needed in extrapolating the proposed formula to different water temperatures and larger velocities, compared to those analyzed in the present study. In the domain of application of Eq. (1), it can be speculated that ${\rm{U}}_{\rm{b}}$ scales with temperature as $\sim {\rm{T}}^{\updelta }$, where δ is, on average, 0.77. Various effects are lumped into this exponent. Overall, for one velocity, the drag force ${\rm{F}}_{\rm{D}}$ may increase by decreasing temperature because the density and the dynamic viscosity of water increase and this leads to increased values of pressure and viscous forces, respectively. This means that, for a given near-bottom velocity ${\rm{U}}_{\rm{b}}$, water viscosity may cause the fish to get tired sooner (i.e. lower ${\rm{t}}_{\rm{ef}}$) at low temperatures than at high temperatures. Furthermore, the temperature exponent is probably dictated by fish-metabolism. In terms of temperature range and related effects on glass eel swimming performance, similar results were found by other authors in the literature. For instance, Harrison et al.^32^ reports that low temperatures (below 10 °C) are known to reduce glass eel activity and that, in general, there is a positive correlation between temperature and upstream-migration speed. Furthermore, it was demonstrated that European eel muscle contractility and efficiency decrease rapidly with water temperature below 10 °C^43,44^. Therefore, low temperatures in rivers may affect eel ecology through both hydrodynamics and physiology, by exerting a direct limiting effect on the movement of the individual. The mechanism controlling the entire breadth of temperatures over which glass eel can have the highest or the lowest swimming performance is still not clear and further research is needed to extend the selected temperature range to achieve comprehensive results for this species. Since European eel is widely distributed across different European climates, we highlight that further investigation can be directed to better determine whether and how water temperature may affect eel swimming performance in different climatic environments and in the context of climate change.

## Methods

### Flume description and experimental setup

**Figure 4:**
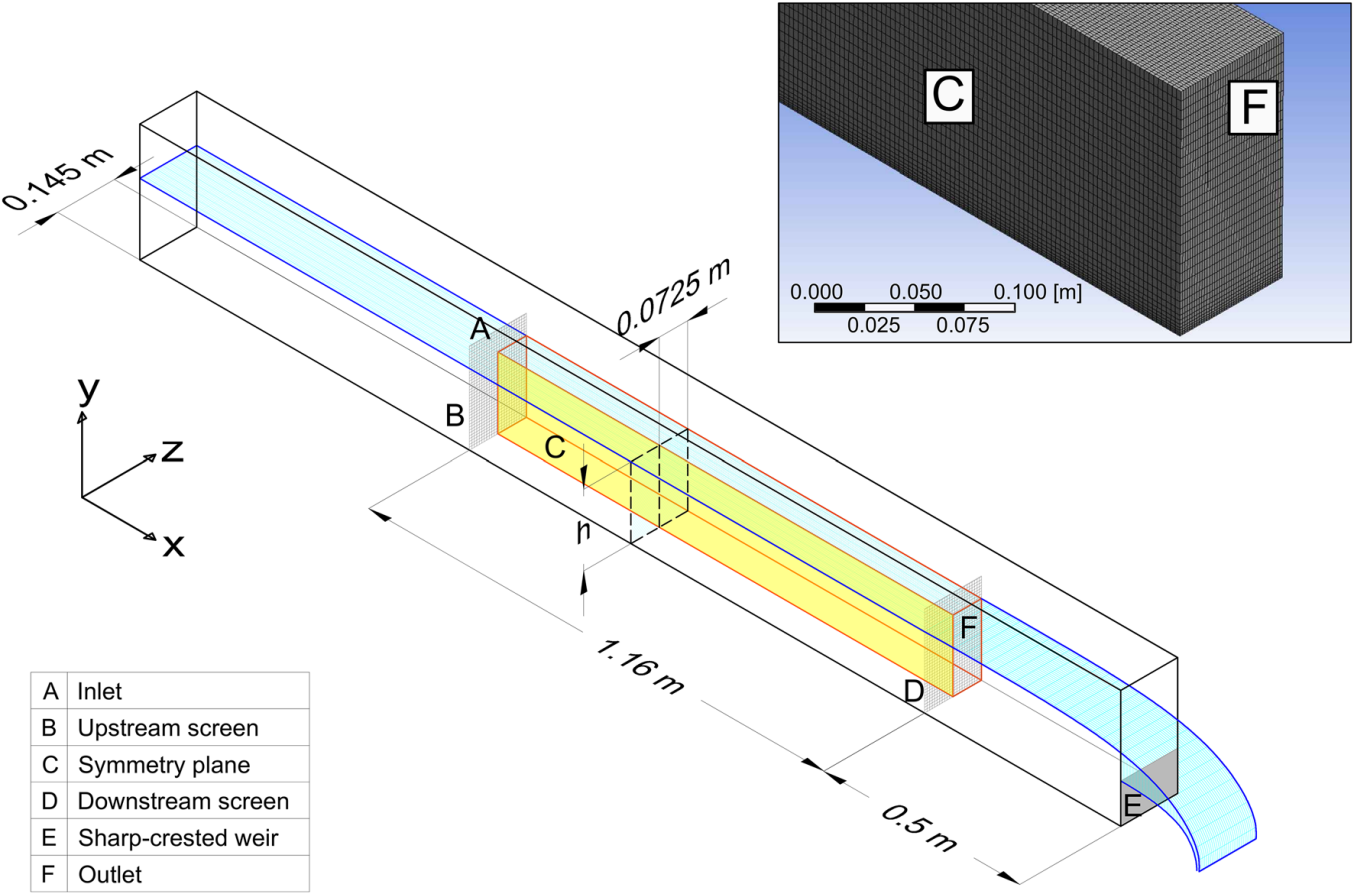
Scheme of the working section of an experimental flume used to estimate glass eel swimming performance at the International Centre for Ecohydraulics Research, University of Southampton. Details on the dimensions of the working section, the mesh size for CFD modelling and the flume components are reported. The letter h represents selected water depth for each experiment to obtain reasonably-uniform flow conditions.

The swimming performance tests were conducted in a hydraulic tilting flume, which was 12 m long, 0.30 m wide and 0.30 m deep. The flume was longitudinally divided in half by a Perspex plate of 1 cm thickness to create two identical channels (0.145 m wide) in which two trials could be conducted simultaneously (Fig. 4). The flume was equipped to control the discharge using a hydraulic pump and a throttling valve. The water depth (*h*), ranging from 0.12 to 0.16 m, and channel slope, ranging from 0.05% and 0.62% were adjusted to create reasonably-uniform flow conditions in the working section of the flume in each trial. The working section, i.e. the volume of water in which eel were left free to navigate, was 1.16 m long and screened up- and downstream by a fine square mesh (1.6 mm). The length of the working section was selected based on experience gained in previous studies^11,23,35^ and the range of average flow velocities selected to cover the threshold between burst and sustained swimming velocities reported in literature. Considering all trials, the flow depth over the entire length of the working section varied by 5 mm at maximum.

Nine different flow conditions (with values of average cross-sectional velocity, U, between 0.15 ms^−1^ and 0.55 ms^−1^) and four different water temperatures (8 °C, 12 °C, 15 °C and 18 °C) were used during the trials (Table 1). Average velocities equal to 0.05 ms^−1^ and 0.10 ms^−1^ were not considered in this study since, at those hydraulics conditions, glass eel could maintain position on the bottom of the channel without actively swimming against the current. Water temperatures were selected as they covered possible European eel critical temperatures for upstream migration in freshwaters^32^. Specifically, Gascuel^45^ and Briand^46^ quoted upstream migration critical temperatures of between 10 °C and 15 °C, whereas in the UK, temperatures of 10–11 °C have been demonstrated as a critical threshold for pigmented elvers ascending weirs or sluices^47^. Trials in which flow velocity was ≥ 0.45 ms^−1^ and water temperature was 8 °C were therefore excluded as the ability of glass eel to swim was greatly reduced and it was impossible to distinguish between un-cooperative behavior and an inability to swim.

**Table 1:** Hydraulic parameters and number of glass eel (eel num.) used in each experiment. For each treatment, represented by an average cross-sectional velocity (U), water depth (D), Slope (S), Reynolds number (Re), Froude number (Fr), experiments were repeated at four different water temperatures (T = 8 °C, 12 °C, 15 °C and 18 °C).

U (m s^-1^)	D (m)	S (%)	Re (–)	Fr (–)	Eel num. T = 8 °C	Eel num. T = 12 °C	Eel num. T = 15 °C	Eel num. T = 18 °C
0.15	0.12	0.05	2.2 × 10^5^	0.14	8	9	10	10
0.2	0.14	0.08	3.1 × 10^5^	0.17	8	9	8	8
0.25	0.15	0.13	3.9 × 10^5^	0.21	8	8	8	8
0.3	0.15	0.18	4.8 × 10^5^	0.24	8	8	8	8
0.35	0.16	0.25	5.7 × 10^5^	0.28	8	8	8	8
0.4	0.16	0.32	6.4 × 10^5^	0.32	8	8	10	10
0.45	0.15	0.41	7.1 × 10^5^	0.37	–	8	10	9
0.5	0.16	0.50	8.1 × 10^5^	0.40	–	8	8	8
0.55	0.15	0.62	8.7 × 10^5^	0.45	–	8	10	8

The Reynolds number is defined as $\rm{Re}=\rm{U}4\rm{R}/\upnu$, where R is the hydraulic radius calculated as the ratio between the wet area and the wet perimeter of the channel cross-section and ν is the water kinematic viscosity. The Froude number is defined as $\rm{Fr}=\rm{U}/\sqrt{\rm{gD}}$, where g is the acceleration due to gravity. Trials in which average velocities U were equal to 0.05 ms^−1^ and 0.10 ms^−1^ were excluded from further analysis because glass eel tended to maintain position on the channel bed and not actively swim against the current. Trials in which flow velocity was ≥ 0.45 ms^−1^ and water temperature was 8 °C were also excluded as it was impossible to distinguish between un-cooperative behavior and an inability to swim.

To investigate different physical parameters of interest (flow velocity, swimming time and water temperature) while maintaining experimental feasibility and statistical rigour, experiments were designed and conducted accommodating pragmatic trade-offs between the number and duration of each trial. For this reason, in each treatment, between 8 to 10 glass eel were tested (279 in total, Table 1) and trials lasted a maximum of 30 min or until the fish fatigued.

### Computational fluid dynamics

In an effort to provide a detailed description of flow field variations within the working section, Computational Fluid Dynamics (CFD) was employed. This technique allowed effective representation of flow velocities experienced by fish in near-wall regions and quantification of the related swimming performance. The CFD model was run using the software ANSYS Fluent (Canonsburg, Pennsylvania, USA). Hexahedral cells of size 10 mm in the center of the domain, refined down with a logarithmic function to 1 mm near the walls, were used to subdivide the domain into finite elements at which hydraulic variables were numerically computed (Fig. 4). The mesh size was estimated to generate mesh-independent outputs and, at the same time, to decrease as much as possible the computational efforts. A k-epsilon (*k-ε*) turbulence closure model was selected to simulate mean flow characteristics. Although it has been shown that the *k-ε* model fails to predict secondary flows in open channels (see e.g.^48^), more sophisticated approaches applied in this study (e.g., Reynolds stress models) showed that the secondary flow vectors did not have a significant impact on the near-wall velocity magnitude. Therefore, to save computational time, the *k-ε* model was employed to simulate all treatment conditions established during the trials. The CFD model also accommodated variations in fluid viscosity and density due to fluctuations in temperature.

At the inlet, the “mass flow inlet boundary condition” was used since the discharge entering the domain was known with adequate precision. Here, the model requires boundary conditions for the turbulent kinetic energy *k* (m^2^ s^−2^) and the turbulent dissipation rate *ε* (m^2^ s^−3^). *k* was computed from direct measurements of velocity fluctuations taken in front of the upstream screen using an Acoustic Doppler Velocimeter (ADV Vectrino-Nortek, Providence, Rhode Island, USA). The turbulent dissipation rate *ε* (m^2^ s^−3^) was estimated as *ε* = *U*^*3*^*/*${l}_{ms}$ using the screen mesh size ${l}_{ms}$ and the bulk velocity U as the characteristic length and velocity scale, respectively. At the downstream edge of the domain, an outflow boundary condition was selected and the free surface was modelled as a symmetry plane. Finally, a longitudinal symmetry plane midway along the flume width was used to decrease the overall computational cost (Fig. 4). All other domain boundaries (sides and channel bed) were set as hydrodynamically smooth walls. The model was validated by comparing CFD results with mean velocity vectors measured by the Nortek’s ADVs (sampling frequency of 25 Hz and point duration of 60 s). ADV measurements were taken at five cross-sections located at a distance of 0.04, 0.29, 0.44, 0.79 and 1.06 m from the upstream screen in each channel. In the center of each cross-section, ADV measurements were taken along a vertical column at 4 locations (i.e., at 0.01 m, 0.03 m, 0.06 m and 0.08 m from the bottom of the channel). Once validation criteria were verified (the velocity profile empirically measured and simulated were significantly similar, t-test p >> 0.10), the simulated flow velocity data were exported from the model and used to estimate the velocity magnitude in the near-wall region.

### Fish capture, holding tanks and experimental procedure

Glass eel were captured during their upstream migration in the Pevensey Haven river, East Sussex, on the night of 18 May 2016 in collaboration with the UK Environment Agency. Fish were transported in chilled and aerated river water to the International Centre for Ecohydraulics Research laboratory at the University of Southampton and held in a flow-through freshwater tank close to the flume. For the first 48 h, water temperature was maintained equal to that of the river at the capture location (12 °C) to facilitate acclimatization and recovery from the initial handling^2^. Glass eel showed a very small degree of pigmentation and very similar body size, with total length ranging from 6 to 10 cm. To exclude fish length effects on the estimate of the swimming performance^10^, only individuals with a total length (TL) between 6.5 cm and 7.5 cm (mean ± SD, TL = 7.1 ± 0.3 cm) were used for this study. The rest of the fish (less than 10% of the total captured) were released back to the river at the capture location. After the period of acclimatization and recovery, water temperature was slowly reduced to 8 °C using chillers with a rate of 1 °C every 12 h. In general, for each experiment, manipulation of water temperature followed the same rate of change and fish were left between 48 and 60 h at the desired temperature, before any swimming tests were conducted. No feeding was carried out during the experiments. The procedure for glass eel capture and hosting were performed in accordance with relevant guidelines and regulations of UK Environment Agency, whereas the entire experimental procure was reviewed and sanctioned by the University of Southampton Animal Welfare and Ethics Review Board.

As the migration of juvenile eel to the tidal limit occurs mainly during periods of darkness (Harrison et al. 2014), all trials were conducted at night at low light levels (0–5 lx). Light level was controlled by an outdoor light sensor adjusted to trigger from 5 lx. Glass eel swimming tests were continuously monitored with the aid of an infrared (IR) video system. Fish swimming behavior was recorded from outside of the channel, in particular from above the free-surface and from the sides through transparent glass walls.

Each glass eel was tested once only. Once the test hydraulic condition was reached, two glass eel were carefully netted from the holding tank and one placed in each of the two flume channels downstream of a plate, enabling fish to shelter from the water current at the beginning of the trial. Infrared cameras (Swann 1080p Bullet cameras, definition1920 × 1080 pixels, 25 frame per second) and IR lighting were switched on when the fish started swimming against the current and the plate was removed. To account for the burst-and-coast swimming behavior the active swimming time of each glass eel was recorded by means of two stopwatches (one stopwatch per channel). When the eel stopped swimming and drifted back with the flow, the watch was stopped; and restarted when active swimming recommenced. The total swimming time, including drift and coast times, was assessed using the recorded time of the IR video system. Due to their largely transparent body, it was not possible to automatically track glass eel trajectories during the trials. However, using videos recorded by IR cameras, it was possible to identify in which spatial volume of the domain eel swam the majority of time (above 90% of active swimming time). In cases where eel became impinged on the downstream screen and ceased moving for more than 15 s, the trial was terminated and the total and active swimming time calculated. Fish that were reluctant to swim were withdrawn from the experiment (0–3.5% per treatment). As the flume was longitudinally divided in half by a transparent Perspex plate, the two individuals could see each other. However, it is reasonable to neglect the vision-induced following behavior, because (i) the light level was very low since experiments were carried out at night, (ii) individuals swam close to each other for less than 1% of the active swimming time on average, and (iii) the resolving power of glass eel eye is quite low (minimum separable angle of 52 min^49^).

### Statistical analyses and construction of fish-swimming curves

Total (t_f_) and active (t_ac_) swimming time were calculated as the median values registered among glass eel tested in each treatment. Fish-swimming curves were constructed using regression analysis to quantify glass eel swimming performance in relation to: (i) the total time t_f_ versus the active swimming time t_ac_; (ii) the average cross-sectional velocity U versus the near-bottom velocity U_b_; and (iii) water temperature T during trials. Swimming curves were expressed in logarithmic scale on both the horizontal (swimming time) and vertical (flow velocity) axes. Therefore, swimming curves of the form of power law (*velocity* = *α time *^*β*^) appear as straight lines in the log–log graph, with the exponent *β* corresponding to the slope, and the constant term *α* corresponding to the intercept of the line^25^.

The analysis of covariance (ANCOVA) was used to compare two or more regression lines by testing the effect of a categorical factor (treatment effect) on a dependent variable in y-axis (y-var) while controlling for the effect of the independent variable in the x-axis (x-var). Regression lines were therefore compared by studying the interaction of the categorical factor with x-var. If the interaction was significantly different from zero, it meant that the effect of x-var on y-var depended on the level of the categorical factor and the regression lines have statistically different slopes. Moreover, when no significant interaction with significant treatment effect was observed, it meant that x-var had the same effect for all levels of the categorical factor, i.e., the regression lines although parallel had significantly different intercepts. The significance level for a given hypothesis t-test was selected as 0.1 for slight significance, 0.05 significance, and 0.01 strong significance of a certain variable.

## Data Availability

The datasets generated during the current study is available via Mendeley Data https://data.mendeley.com/datasets/bwrnz25tbc/1 (10.17632/bwrnz25tbc.1).
